# Simian Selves: From the Mirror Test to Habeas Corpus

**DOI:** 10.3390/ani16152355

**Published:** 2026-08-02

**Authors:** Shira Shmuely

**Affiliations:** Max Planck Institute for Comparative and International Private Law, 20148 Hamburg, Germany; shiras.mail@gmail.com

**Keywords:** history of science, animal law, animal welfare, primates, legal personhood, self-awareness, mirror self-recognition (MSR)

## Abstract

This paper traces the historical transformation of the scientific and legal conceptions of nonhuman primates’ minds from the nineteenth century to the present, focusing on the emergence of self-awareness as a criterion for legal protection. It argues that scientific developments reshaped animal law by shifting attention from animals’ capacity to suffer toward their capacity to possess a self. Drawing on scientific publications, legal documents, and animal rights litigation, this paper examines how experimental approaches to simian cognition, especially Gordon Gallup’s mirror self-recognition (MSR) test, became foundational evidence in campaigns for nonhuman legal personhood. This study centers on the 2013 habeas corpus petition filed on behalf of the chimpanzee Tommy by the Nonhuman Rights Project, showing how scientific claims about chimpanzee cognition entered the courtroom through expert affidavits. By situating these legal developments within longer histories of primate research, colonial extraction, and debates over species difference, this paper demonstrates that the scientific and legal definitions of the chimpanzee and of the legal subject were redrawn together. The rise in cognitively grounded animal rights does reproduce hierarchies based on perceived proximity to humans, as critics have argued. But the figure against which that proximity was measured was itself being remade at each turn.

## Simple Summary

What makes a being deserving of legal rights? Is it the ability to feel pain or the capacity to know oneself as an individual? This study traces how shifting scientific answers to these questions have shaped Western animal law over the last two hundred years, focusing on nonhuman primates. In the UK, the first laws protecting animals emerged in the nineteenth century. They focused on an animal’s capacity to feel pain rather than its mental abilities. This research follows the historical shift toward recognizing self-awareness as a new standard for granting legal rights. It analyzes how experiments like the mirror self-recognition test, which demonstrated that chimpanzees can recognize their own reflections, became evidence in the 2013 legal case seeking personhood for a chimpanzee named Tommy. By situating this landmark case in the history of human–animal relations, this paper suggests that the scientific and legal definitions of “human” and “animal” change together over time. Emphasizing similarities to humans has helped advocate for the rights of some primates. However, it also risks creating new hierarchies that leave other species behind. This research clarifies the role of scientific knowledge in shaping our ethical and legal responsibilities toward other living beings. It helps us understand the evolving boundaries of multispecies justice in a changing world.

## 1. Introduction

In December 2013, a chimpanzee named Tommy became the first nonhuman animal in U.S. history to be represented in a habeas corpus hearing, challenging centuries of legal precedent that treated animals mainly as property. This landmark case emerged from the Nonhuman Rights Project (NhRP), a litigation organization established in 1995 by American lawyer Steven Wise (1950–2024). Although by 2026 the NhRP had expanded its advocacy to include elephants and dogs, most of its clients have been chimpanzees, selected specifically for their documented cognitive capabilities. As Wise explained, “because Jane [Goodall] and others have studied chimpanzees intensively for decades, we know the extraordinary cognitive capabilities that they have, and they also resemble the kind of [cognitive capabilities] human beings have” [1].

Wise’s statement encapsulates a profound historical shift in the ethical foundations of animal law. Before the 1970s, animal protection movements in the U.S. and the UK primarily emphasized a species’ capacity to feel pain, rather than mental abilities, as justifying legal protection. This approach drew upon the tradition of utilitarian thought, which dominated debates about the moral status of animals for about two centuries. A species’ sensitivity to pain was the major justification for providing some restrictions on its use, and policies regarding animal use, at least in theory, drew upon a calculation balancing the desirability of the objectives of the pain-inducing act for humans, with the amount and duration of animal pain [2]. Beginning in the 1970s, however, science-based claims about animal cognition gained increased prominence in philosophical and legal arguments for animal rights. This transformation is epitomized in the litigation advanced by the NhRP. Understanding the shift from animals’ sensitivity to pain to animals’ minds requires unraveling the expanding role of science in animal ethics and the movement of knowledge across field research, laboratory experiments, and courtrooms.

Drawing on scientific publications and legal documents, this paper shows how developments in primatology, psychology, and brain science converged with animal ethics to fundamentally reshape animal law. This paper concerns shifting justifications for granting legal protections to animals from their showing sensitivity to pain to demonstrating a self, understood as a subject aware of its own existence. This move was influenced by what is commonly referred to as “the cognitive revolution” in the sciences: a move away from behaviorist approaches toward the acknowledgement of internal mental processes. The idea, which gained momentum in the mid-twentieth century, was to bring the human thought itself within empirical reach [3,4,5]. Twentieth-century attempts to gain access to the inner processes of great apes and other animals resulted in a set of tests and experimental procedures which founded a specific idea of what it means to be aware of oneself. These tests proved instrumental for twenty-first-century advocacy for widening the scope of legal protection to nonhuman species who demonstrate those characteristics.

Although this study provides examples from Europe, the UK, and the United States, scientifically informed animal rights litigation is not unique to these jurisdictions. In 2015, for example, a Buenos Aires court recognized the orangutan Sandra as a nonhuman person based in part on scientific evidence of her cognitive abilities, ordering conditions to preserve those abilities. A year later, another court in Argentina granted a habeas corpus writ to chimpanzee Cecilia, held by Mendoza Zoo [6,7]. In 2017, a chamber of Colombia’s Supreme Court granted habeas corpus writ to an Andean bear named Chucho (later overturned) [8]. While formed in different legal cultures and social settings, these cases demonstrate the transnational qualities of contemporary animal law and flesh out the movement of moral ideas and scientific knowledge across continents [9].

While existing scholarship has richly documented the evolution of social attitudes toward animals, analyzing among others the roles of economy, religion, nationalism, and class, the role of scientific knowledge about animal cognition in driving these changes has received less attention. And while the history of animal cognition science is itself increasingly studied, its uptake in ethical and legal reasoning has not been systematically examined. As this paper shows, starting in the mid-twentieth century, ethicists and policymakers cited primates’ cognitive abilities as justification for legal protections and drew on them to advance theories of animal rights. This history adds an important aspect to the growing literature examining the extraction and circulation of simians’ bodies in the production of biomedical knowledge and highlights the role great apes played in the conceptualization of consciousness.

The history offered here speaks to recent critiques of the animal rights project. Scholars have argued that the strategy of recognizing personhood for species which resemble humans reproduces a humanist legal framework that leaves other animals invisible [10,11,12]. The historical material developed below largely bears this critique out. A high-stakes fascination with sameness and difference has structured the relations between the species since the eighteenth century. Yet the same history also complicates the critique. Those critical accounts tend to treat the human as a stable referent in law, science, and public imagination, while the conceptualization of nonhuman species, the chimpanzee in this case, shifts continuously, included or excluded from the moral calculus according to perceived resemblance to human bodies and mental capacities.

What the long history of the simian self suggests is that the successive criteria for animal inclusion, from brain anatomy to language to mirror self-recognition, have emerged at moments when the scientific figure of the human itself was being redrawn. The boundary moves on both sides at once. This history helps us understand not only why the legal status of nonhuman primates changed through the twentieth century, but it also provides us with analytical tools to approach, for example, the recent inclusion of invertebrates in UK animal welfare legislation [13]. The changing ideas about animals’ inner experiences, their cognitive and mental capacities, lead to reconsiderations of which species should be protected by law and how.

The first section follows the developments in primate research from nineteenth-century comparative anatomy and its focus on brain structure to twentieth-century field primatology, home observations, and laboratory research. This section focuses on American psychologist Gordon G. Gallup and his mirror self-recognition (MSR) experiments, which would become instrumental in animal rights litigation four decades later. The second section provides an overview of the status of nonhuman primates in animal law, showing how emerging legal discourse concerning primates has been heavily informed by scientific findings about their “human-like” traits and associated cognitive capacities. The third section analyzes the integration of scientific knowledge and legal reasoning, as demonstrated in Tommy’s case. The scientific preoccupation with the animal mind, and particularly the simian mind, sets the conditions for the move from animal welfare to animal rights. The departure from pain-centered ideas about animals necessitated a new legal mechanism that crystallized in the habeas corpus petitions of the NhRP. Together, this analysis shows how scientific knowledge, legal argumentation, and ethical claims about simian minds co-emerged in the second half of the twentieth century, introducing a new terminology, a new set of experts, and new sets of claims into the courtroom.

## 2. Materials and Methods

This study employs a historical and interdisciplinary legal analysis to trace the evolving conceptualizations of nonhuman primate minds from the nineteenth century to the present. The research materials consist of a diverse corpus of primary sources, including nineteenth-century comparative anatomy publications, mid-twentieth-century laboratory research, and contemporary legal documents. Specific focus is placed on the published records of Gordon Gallup’s mirror self-recognition (MSR) experiments and the case files from the Nonhuman Rights Project’s (NhRP) 2013 habeas corpus petition for the chimpanzee Tommy. These materials were analyzed to identify the movement of scientific knowledge, such as data on self-awareness, empathy, and future planning, from the laboratory into the courtroom.

## 3. Results

This research identifies a profound transformation in the ethical and legal foundations of animal protection, moving from a utilitarian “pain-based” model to a “cognition-based” framework. This study reveals that the scientific and legal definitions of the subject are co-constructed; as criteria for animal inclusion (such as MSR) are forged, the legal and social definitions of the “human” are simultaneously redrawn.

## 4. Discussion: From the Anatomy of Difference to Great Apes as Proxies for the Human Mind

Simians, extracted in increasing numbers through European imperial networks in Africa, South America, and Southeast Asia, played a key role in eighteenth- and nineteenth-century taxonomical studies and brain research, especially in comparative anatomy and localization experiments. During this period, scientific and cultural interest in the anatomical proximity between humans and apes carried deep metaphysical and racial significance [14,15,16]. Nonhuman primates were also prominently featured in European and American zoos, where they were presented as spectacles that blurred the boundary between animal and human society. Scholars had closely examined how in both scientific discourse and public display, apes often symbolized colonized peoples, their representations saturated with racial and gendered meanings [17,18,19,20].

The simian brain attracted special interest from the very first encounters of European naturalists with the bodies of great apes, as demonstrated in the works of seventeenth-century British physician Edward Tyson [21] and eighteenth-century Dutch anatomist Pieter Camper [22]. But it was in the boiling moments of mid-nineteenth-century evolution debates that this fleshy organ became the focal point for arguing over the place of human beings in the world of living beings. The “hippocampus controversy” between British biologists Thomas Henry Huxley and Richard Owen exemplified the centrality of the brain in demarcating humans from other animals. Drawing upon his dissection of a gorilla, Owen contended that he could pinpoint the anatomical difference between human and nonhuman brains to the former’s hippocampus minor (calcar avis) and two additional related structures. This form, argued Owen in 1859, was linked to “peculiar mental powers,” and on the basis of this alleged finding, he was “led to regard the genus Homo as not merely a representative of a distinct order, but of a distinct sub-class of the Mammalia” [23] (p. 26). However, Huxley, Owen’s bitter rival on many fronts, showed the existence of the same features in the brains of apes and other primates [24].

The unsettling anatomical similarity in brain structure directed those who struggled to locate the difference between the species to the less measurable terrains of emotions and cognition. Here, the associations among the species were more pliable. Historian Harriet Ritvo explained that some nineteenth-century thinkers sought to “displace apes from their awkward proximity” to humans. For instance, George Romanes, a biologist and a close associate of Darwin, suggested that dogs rather than apes were the closest to humans in their mental capacities [25]. Researchers into animal mental capacities, therefore, have not always been crowning great apes for having a peculiarly human-like mind. The special status of nonhuman primates, which seems intuitive to contemporary readers, had developed through the nineteenth century and was crystallized in the following century.

The unattainable task of locating the definitive difference between human and nonhuman minds in the flesh confronted nineteenth-century European anatomists not only with theological conundrums but also with the boundaries of their power to know. In other words, it was not only humans’ spiritual superiority that came under doubt, to which some of them could adhere, but also humans’ cognitive preeminence—an uneasy realization for children of the Enlightenment. Historians Fiona Coll and Jennifer Esmail maintain that Victorian scientific and popular accounts disclose the “inability to encounter directly an ape’s mind,” thereby manifesting “the limits of human cognition” [26]. Finding a way to examine the inner lives of animals was therefore required not only for situating humans in evolutionary scales. It was also necessary as it was demonstrative of the superb capacities of humans, endowed on them by the alleged cognitive progression of the species.

The steadily increasing supply of living simians to laboratories, zoos, and research stations provided the opportunity to systemize research into simian minds [27,28]. Colonial and postcolonial networks worked tirelessly through the twentieth century to deliver various monkeys, notably capuchins and rhesus macaques, and great apes, mainly chimpanzees, for biomedical projects such as pathological research and vaccine development [29,30,31]. The extraction and forced movement of simians from South America, Africa, and South Asia to Europe and North America not only shaped Western biomedical knowledge and enriched its industry. The engagement with and the observation of other simians were also constitutive in the conceptualization of human societies and group behavior among European and American scientists. Historians of science show how primate research perpetuated ideas such as race and class [32], gendered aggression [33,34], parenthood [35], and culture [36]. A feminist turn in primatology brought attention to elements of cooperation, positioning ape societies as alternative models for understanding human social and political life [37].

However, the contribution of primates to the history of science went beyond being test tubes for disease research or representations of Western political fantasies. Captive simians also played a crucial role in shaping scientific ideas about the mind, the self, and the very essence of being a conscious subject.

## 5. Gallup’s Mirror: The Mark Test and the Simian Self

Humans watch nonhuman animals. Animals observe humans and each other. But can animals look at themselves as subjects, as selves? Chimpanzees were the first to show scientists that they can in a set of experiments that would become pivotal to animal rights litigation. In the 1960s, American psychologist Gordon G. Gallup noted that most of the animals he observed react to their reflection in the mirror as though they were confronted with another organism. For example, they demonstrated aggressive behavior or approached their reflection as a playmate. Others ignored the mirror altogether. However, some exhibited self-directed behaviors, and Gallup notably mentioned one chimpanzee who groomed in front of the mirror the otherwise hidden parts of his body [38].

These initial observations drew on a well-established experimental repertoire. Historian Katja Guenther showed that mirrors had served as tools for examining mental development since the nineteenth century. The method, which proved useful for overcoming the lack of language in infants and in inarticulate individuals, was refined through various twentieth-century research protocols [39]. In 1970, Gallup introduced a crucial modification to the mirror test in the landmark publication, “Chimpanzees: Self-Recognition” [40]. This experiment was conducted on four wild-born chimpanzees in Delta Regional Primate Research Center. The researchers isolated the chimpanzees in separate small cages for two days, after which they placed a mirror in front of the cages. The chimpanzees’ responses to the mirrors were closely documented. After ten days of being accustomed to the presence of the mirror, the chimpanzees were anesthetized, and the researchers placed a mark above one eyebrow and behind one ear with an odorless, nonirritating red dye. The recovered chimpanzees attended to the marks that were only visible through the mirror, and Gallup celebrated “the first experimental demonstration of a self-concept in a subhuman form” [40] (pp. 86–87). This research ultimately aimed to understand the evolution of human cognition, but in the following decades, the emphasis would shift to express curiosity about the way other living beings experience themselves. A new experimental path into the inner lives of simians was established through anesthetics, some paint, and a mirror.

Self-recognition, according to Gallup, means “being able to be the object of your own attention” [41]. This definition appears to describe a solitary experience, but Gallup’s experiments have demonstrated that the capacity for self-recognition depends upon social exposure. He showed this by experimenting with the mark test on captivity-born chimpanzees who had been separated from their mothers soon after birth to be reared isolated from social interaction. The results showed that, unlike wild-born chimpanzees or captive chimpanzees exposed to their conspecifics, “chimps reared in isolation showed no evidence of being able to recognize themselves in mirrors” [42] (p. 73). Early social experience was therefore shown to be a precondition for self-awareness.

An important aspect of mark test experiments is their discriminatory classificatory power, as they provided a new way to rank organisms according to their mental capacity. Already in 1970 Gallup noted a “decisive difference between monkeys and chimp” regarding their response to the mark on their foreheads [42] (p. 87). And while Gallup demonstrated that having a concept of self is a socially dependent acquired phenomenon, he rejected claims made by other scientists for a similar behavior in species other than chimpanzees and orangutans, arguing that there were “phyletic limits of this capacity” [43] (p. 329). Gallup’s viewpoint insisted that the faculty of self-recognition “might be limited to those primates most closely related to humans” [44] (p. 325).

Gorillas, however, did not square with the theory of the distribution of self-awareness capacity. Study after study, they showed no self-directed actions when faced with their own reflection. For example, in 1981, experimenters tried the mark test on two adult gorillas, both of whom had been captured in the wild and purchased by Houston Zoological Gardens as infants from a private dealer about ten years earlier. While both animals spent time viewing the mirror, the experimenters observed no mark-directed responses [45]. Home-reared gorilla Koko, enlisted in a language acquisition program led by psychologist Francine Patterson, was one documented exception [46] (p. 273). This raised an evolutionary puzzle: how could humans, chimpanzees, and orangutans share a cognitive capacity that gorillas appeared to lack, despite gorillas’ more recent divergence from the human–chimpanzee lineage than orangutans?

Biologist Daniel J. Povinelli proposed that mirror self-recognition might represent an ancestral trait that can reappear under specific developmental conditions. Gorillas, according to this explanation, “turned off” this trait over the course of evolution. Povinelli suggested that the intense tutoring Koko underwent produced a “profound disturbance” in her development, resulting in the re-emergence of an ancestral characteristic. This, he emphasized, was not directly attributable to Koko’s language training “but rather aspects of cognitive stimulation related to the construction of a sense of personal agency” [47] (pp. 296–297). In this interpretation, Koko’s sense of self was buried by evolution, only to be accidentally evoked by her human trainer.

Xebo’s case moderated the significance of humans in sparking gorillas’ self-awareness. He was born in Rotterdam Zoo in 1985, and unlike Koko, he was raised in the company of conspecifics at Barcelona Zoo. According to researchers Sandra Posada and Montserrat Colell, he nevertheless demonstrated mirror self-recognition. They argued that this capacity stemmed from Xebo’s psychological wellbeing and environmental enrichment, conditions they characterized not as pathological disturbances but as appropriate development [48]. What is telling about the observations of Koko and Xebo is that they advanced a view of the emergence of signs of self-conception in the context of social interaction, whether through an intra- or interspecies engagement.

Over subsequent decades, researchers reported with different levels of assurance on mirror self-recognition in aquatic mammals such as bottlenose dolphins, birds such as the Indian house crow, fish such as the cleaner wrasse, and even invertebrates such as the ghost crab [49,50,51,52,53]. In light of these studies, scholars, including primatologist and ethologist Frans de Waal, challenged the binary perspective on self-awareness and called for the consideration of “a gradualist model of the various ways in which animals construe a self” [54]. But Gallup remained unconvinced. Half a century after the initial experiment, Gallup still claimed that only great apes, and specifically chimpanzees and orangutans, exhibited clear, consistent, and replicable behaviors of self-recognition [55]. In this view, the difference between the species is of kind rather than degree; there is no gradual evolution of self-recognition but rather an emergence of it.

The mark test, designed to bypass the problem of cross-species communication, emerged during the high point of ape language research in the U.S. The 1970s, which historian Amanda Rees has described as a “renaissance in primate cognition studies” [56] (p. 165), saw multiple institutionally funded projects explicitly aimed at testing whether nonhuman primates could acquire language in a human sense. Although working in different fields, researchers engaged in MSR and in linguistic development cross-referred to each other’s publications. Gallup cited Keith and Cathy Hayes’ studies with Vicki [42] (p. 74), and Patterson and Cohn examined Gallup’s MSR experiment on Koko [46] (p. 274). In addition to the mirror self-recognition test and language acquisition research, the scientific grounding of human–ape similarities saw a proliferation across multiple disciplines, including genetics. The April 1975 cover of *Science* featured the influential publication of geneticist Mary-Claire King and biologist A. C. Wilson, who argued for 99% identity in coding the protein genes of humans and chimpanzees [57]. Primatologists Shirley Strum and Linda Fedigan noted that in the mid-1980s, the animal mind also became a central concern in field research [58].

In subsequent decades, the mark test for self-recognition drew various critiques ranging from challenges to its validity as evidence of self-awareness [59] to arguments that its application is too narrow in terms of relevant species [60]. One such critique is that it is a visual-based test, while many creatures experience the world, and supposedly themselves, through other senses. Scholars have therefore suggested alternative ways to create a species-relevant test analogous to mirror self-recognition, such as an odor test to replace the mirror when examining dogs’ self-recognition [61]. Whether in its classical form or in subsequent variations, the mark test became central scientific evidence for self-awareness.

As data accumulated, it became evident that even within chimpanzees (the paradigmatic species for self-recognition), the rates of “passing” the self-recognition test varied across individuals. The mark test thus performed a double operation. It destabilized the human–animal boundary by demonstrating through scientific methods that some nonhuman animals possess self-awareness. Simultaneously, it established a new classificatory device, dividing those capable of self-recognition from those forever excluded from it—initially across species and later also among individuals. This duality was replicated once the mirror test had been incorporated into claims for animals’ legal personhood.

## 6. Primate Cognition in Twentieth-Century Law

The accumulated knowledge about primates’ mental lives was harnessed to make claims for their moral status. Instances of special legal protections for great apes based on their distinctive cognitive abilities appeared in regulations overseeing animal experimentation in various jurisdictions in Europe, the UK, the United States, and beyond.

In 1985, the U.S. amended the federal Animal Welfare Act (AWA) of 1966 to set standards “to promote the psychological well-being of primates,” singling out primates for psychological consideration under this legislation. A subsequent experts’ workshop gathered in December 1987 to draft recommendations for the United States Department of Agriculture (USDA) tailored for chimpanzees. The final document stressed that chimpanzees possessed “many cognitive abilities once viewed as uniquely human,” among which are “reasoned thought, generalization, abstraction, symbolic representation, and self-awareness.” Moreover, these “sibling species” are entitled to “special treatment”:

“It is precisely because chimpanzees are so like humans physiologically that they are used by medical researchers in the attempt to alleviate human suffering and illness. And it is also because they are so like ourselves emotionally, cognitively and behaviorally that we must improve the quality of their lives during those long years of imprisonment and servitude” [62] (pp. 278–279).

Other U.S. policies reflected a gradual retraction from using great apes in biomedical research. In June 2015, the U.S. Fish and Wildlife Service classified all captive chimpanzees as endangered under the Endangered Species Act (ESA), ending the earlier split listing that had designated wild chimpanzees as endangered but captive ones as threatened. The change formed part of a broader policy shift away from chimpanzee experimentation [63]. In November 2015, the National Institutes of Health (NIH) announced that it would no longer support biomedical research on chimpanzees and would retire all NIH-owned and NIH-supported animals to sanctuaries.

In the UK, a 1981 report by the Advisory Committee on Animal Experiments recommended affording special protection to primates by administrative means [64]. The Animals (Scientific Procedures) Act 1986 (ASPA) stipulated that experimenters who wished to operate upon primates, similarly to those who planned to experiment on cats, dogs, and Equidae, were required to explain why no other species was suitable for the purpose of their research. Great apes have not been used in UK experiments since the 1986 Act, and a ban on the experimental use of great apes came into force in November 1997 [65]. Great apes were not the only primates to receive special consideration. In 2006, a working group sponsored by The Academy of Medical Sciences, the Medical Research Council, The Royal Society, and the Wellcome Trust noted that public concern about the use of nonhuman primates in general in research significantly exceeded concern about other animals [66] (p. 12). The report also held that nonhuman primates “are generally considered to be more intelligent than other mammals, although the criteria for defining intelligence are a constant source of debate” [66] (p. 63). For comparison, in a rare 1881 case court involving nonhuman primates, the British anti-vivisection organization Victoria Street Society charged British neurologist David Ferrier for violating animal experimentation regulations. While Ferrier explained in his scientific publication that he experimented on monkeys which “most resemble man” [67], the court discussion did not give special consideration to the species involved [68].

In New Zealand, the Animal Welfare Act 1999 requires special administrative approval for “research, testing, or teaching involving the use of a non-human hominid” [69]. Activist Rowan Taylor traces the beginning of the campaign for great apes’ rights in New Zealand to 1980, noting “research findings whose philosophical implications had been noted by Gordon Gallup, Jr. and Carl Sagan among others” [70] (p. 36).

Similar concerns appeared in EU regulation. Guidelines from 2007 for caging nonhuman primates state that, notwithstanding their wild nature and climbing habits, “non-human primates have advanced cognitive capabilities and complex foraging and social behaviour” [71] (p. 37). No such remarks were made regarding other species. The special status of great apes was emphasized also in Directive 2010/63/EU of the European Parliament and of the Council of 22 September 2010 on the protection of animals used for scientific purposes. Section 17 of the Directive refers to the “specific ethical and practical problems” arising from great apes’ genetic proximity to human beings and their highly developed social skills. The directive subjects the use of nonhuman primates to restrictions and prohibits (with exemptions) the use of great apes in scientific procedures [72].

These examples show how, starting roughly in the 1980s, scientific characterizations of cognition and sociality functioned as justifications for the differentiated legal treatment of nonhuman primates and specifically great apes. In the instances described above, however, the argument was mostly made to improve the conditions under which primates were held and used in experimentation. In other words, the cognitive proximity of nonhuman primates to humans served as reasons for mitigating their conditions of confinement within the tradition of animal welfare law. In these same decades, ethicists and animal researchers have been developing the argument that the scientific insights into simian cognition should lead to a fundamental reassessment of their moral and legal status. From their perspective, the animal welfare system was inadequate to capture what was being valued, namely the simian mind: regulations operating within the framework of animal welfare could ostensibly monitor the levels of animal pain, but they could not effectively provide for a mental concept such as self-awareness. Put differently, the older system, which was constructed to alleviate concerns regarding the pain of animals, was ill equipped to provide a legal safeguard to the animals’ realization of subjective self.

## 7. The Mirror in the Courtroom: Self-Recognition as Legal Evidence

The call for rights for great apes was advanced forcefully by the globally influential Great Ape Project. Launched by philosophers Peter Singer and Paola Cavalieri with an essay anthology bearing this title in 1994, the organization brought together ethicists and animal researchers from a variety of fields. This project was driven by the accumulating scientific findings about the minds of great apes. As its patrons explained, “we have now sufficient information about the capacities of chimpanzees, gorillas, and orang-outans to make it clear that the moral boundary we draw between us and them is indefensible.” In this political philosophy, the membership within the moral community should be grounded in the fact that humans “are intelligent beings with a rich and varied social and emotional life,” and therefore other species who exhibit the same capacities should be included too. The Declaration of Great Apes enclosed to the essays stipulated again that these creatures “have the mental capacities and the emotional life sufficient to justify inclusion within the community of equals.” The Declaration calls for the acknowledgment of three principles: the right to life, the protection of individual liberty, and the prohibition of torture. While the third principle refers to unnecessary suffering, the first two have nothing to do with pain. They reframe the value of great apes as lying in their mental experience of the world as selves [73] (pp. 1–7).

The Great Ape Project was an interdisciplinary endeavor bridging between scientific research into simian minds and ethical commitments. It is in this intellectual atmosphere that Steven Wise, an animal law practitioner, founded in 1995 the NhRP [74]. Wise charted the legal vision undergirding the organization in the book *Rattling the Cage*, where he stated, “minds are critical for legal rights” [75]. Several of the primatologists cited in Wise’s book—Sue Savage-Rumbaugh, Tetsuro Matsuzawa, and Christophe Boesch—would submit supportive affidavits to Tommy’s case thirteen years later. American Justice Richard Posner criticized Wise’s vision and contended that existing legal doctrines, such as property, were not only strategically useful but also ethically compatible with caring for animals, since “people tend to protect what they own” [76] (p. 539). Posner accurately predicted that common law judges will render animal rights litigators’ ideas revolutionary and relegate the decision about animals’ basic rights to legislators. Despite this critique and others like it, Wise moved on with the first habeas corpus petition.

Tommy was the subject of the first attempt to turn this animal rights theory into a litigation case and the scientific mirror self-recognition test into legal evidence. The petition was submitted to the Supreme Court of Fulton County, New York, by Steven Wise and attorney Elizbeth Stein. The common law writ of habeas corpus empowers the courts to demand that a detaining authority justify a person’s confinement. If the confinement is unlawful, the court can order release. The argument of the NhRP was that there is no fixed definition of “legal person.” In various other contexts, the U.S. law acknowledged nonhuman entities, including corporations, as legal persons. Therefore, the NhRP argued that this definition should extend, for the purpose of habeas corpus, to nonhuman animals. Specifically, it should extend to those individuals who demonstrate autonomy such as Tommy [77]. The litigators knew little about Tommy’s biography: a male estimated to have been born in the early 1980s, he was allegedly employed in the film industry before reaching the cage on State Highway 30, where his only companion was a TV screen. Naming Tommy as a client in the petition was a powerful rhetorical move. It at once narrowed the legal claim to a single creature and destabilized a system in which animals are treated in blocks. The focus on one individual belongs not only to a history of celebrity animals who entertain the public against the background of mass use [78] but also to an ongoing effort to tell the singular life stories of animals and acknowledge their individuality [79,80,81,82].

As legal scholar Anne Peters explains, “the broad function of personhood is to identity and circumscribe membership in a (spiritual, social or legal) community, and concomitantly who gets the benefits going with membership” [83] (p. 432). Peters goes on to argue that animal personhood is a precondition to animal rights. The path to animal rights goes therefore through animal personhood, and the reliance on scientific evidence was an explicit strategy by the NhRP. The organization annexed its early petition on behalf of Tommy with nine affidavits signed by experts combining field and laboratory studies including primate behavior, comparative psychology, ecology, evolutionary primatology, and cognitive zoology. The experts’ opinions covered areas such as chimpanzees’ material culture and use of tools, emotional expression, communication skills, future planning, personality, and intelligence [84,85,86,87,88]. 

For the NhRP to make the habeas corpus claim, and for the court to acknowledge Tommy’s right to liberty, the chimpanzee first had to be constituted as an individual capable of valuing autonomy. Legal personhood was thus anchored in Tommy’s capacity for self-recognition. It is unknown whether Tommy ever held a mirror in his hand, but litigators inferred his self-awareness from knowledge generated about the species to which he belonged. The petition’s affidavits grounded this personhood in Tommy’s capacity for self-recognition, treating it as a baseline from which social and temporal life emerged. Primatologist Tetsuro Matsuzawa associated selfhood with the capacity for goal-directed action: from self-recognition stems the ability to form, pursue, and evaluate desires [88] (p. 6). Psychologist James R. Anderson noted a link between chimpanzees’ mirror self-recognition and their empathic abilities. He contended that identifying oneself in the mirror builds upon the ability to create a mental representation of the image of oneself from another visual perspective. This capacity is essential to empathic feelings [89] (p. 4). Primatologist Christophe Boesch opined about chimpanzees’ autobiographical self, arguing that chimpanzees are self-aware and understand that they exist through time, and thus they can relate mentally to past and future events. Moreover, Boesch contended that self-recognition extends to having a concept of death as an irreversible situation [90] (pp. 6–7).

What was missing from the experts’ scientific overviews of chimpanzee selfhood was the considerable research showing that early social interaction is a precondition for mirror self-recognition, as shown in comparative studies of socially isolated chimpanzees. Whether this evidence was overlooked, dismissed as unimportant, or deliberately omitted, the resulting narrative emphasizes innate, stabilized, and individualized conceptions of self. This framing may align with the legal construction of personhood better and allowed the litigators to refer from species-level MSR research to Tommy as an individual client. However, the individualized concept of the self sacrifices an important dimension of animal mental life, one that casts a critical light on the conditions of captivity: there is circularity about demanding that animals exhibit a trait whose appearance depends upon the very rearing conditions of which they have been deprived.

The Fulton County Supreme Court Justice, Joseph M. Sise, expressed his sympathy for the case but denied the petition without extensive elaboration. In December 2014, the Appellate Division of the Third Judicial Department rejected the NhRP’s appeal. It ruled that “the ascription of rights has historically been connected with the imposition of societal obligations and duties” [91] (p. 4). In other words, to enjoy rights, one must bear duties, and therefore chimpanzees could not be considered legal persons. The foundations of legal rights, according to this court decision, have more to do with social functioning than with innate mental capacities. Subsequent attempts to appeal were unsuccessful.

In December 2015, the NhRP filed a new habeas corpus petition on Tommy’s behalf, this time emphasizing that chimpanzees can have and bear duties and responsibilities towards other chimpanzees and also towards humans while also contending that duties are not a prerequisite for rights [92]. The NhRP used experts’ affidavits to tie together chimpanzees’ complex cognition and their social ties. In this way, the organization attempted to bring back scientific evidence into the discussion which the courts stirred towards abstract legal theory. But the court declined to debate these claims substantially, arguing that there was no justification for revisiting the prior decision. Tommy’s petition was followed by various legal procedures, including in one point the consolidation of this petition with another petition on behalf of the chimpanzee named Kiko. In June 2017, the Appellate Division of the New York Supreme Court, First Department, affirmed previous decisions to decline the orders of habeas corpus. It contended that there was no indication that American law makers intended to protect nonhuman animals’ rights to liberty and that “the asserted cognitive and linguistic capabilities of chimpanzees do not translate to a chimpanzee’s capacity or ability, like humans, to bear legal duties, or to be held legally accountable for their actions” [93] (p. 3). Here the court flipped the species’ logic that the petitioners presented: the litigants, as explained above, argued that their claims were focused on Tommy and him alone and that they do not request the release of all animals, not even all chimpanzees. The Appellate Division court, however, based its decision on the species level. It explained, for example, that while infants and comatose persons do not bear duties, “these are still human beings, members of the human community” [93] (p. 3). In 2022, Tommy died, a fact the NhRP discovered only a year later, and this brought the endeavor to his release to its halt.

Legal scholar Kristen Stilt shows how while the judge attempted to steer the discussion towards improving the confinement conditions of Tommy, Wise insisted upon the removal of the chimpanzee from its confinement [94]. In none of the decisions, however, was the science which grounds the claim questioned. The courts seemed willing to accept scientific evidence of a “self,” but they remained resistant to granting “rights” if that self cannot function as a duty-holding subject in human society. Even publications which fundamentally oppose animal rights claims such as the commentary by lawyer Michelle Pardo acknowledge the strengths of existing scientific evidence, yet she adds that the changing nature of science destabilizes the claims [95]. It is too early to evaluate whether the NhRP’s approach that interwinds legal personhood and scientific evidence on subjecthood was successful in gaining its own objectives or not. Its significance lies in challenging the legal system to move beyond traditional welfare models and address the animal as a subjective self.

## 8. Conclusions

Scientific conceptions of simians’ minds have shifted over the past two centuries, from an emphasis on anatomical differences between human and nonhuman primates to a focus on shared cognitive and emotional traits. Findings about the minds of great apes translated into ethical claims and legal action in the last few decades of the twentieth century, culminating with path-breaking petitions for the habeas corpus of chimpanzees and other species. Responding to these latest developments, legal scholar and ethnographer Irus Braverman calls for replacing the “typically reformist discourse of animal rights.” In this view, part of the problem of the animal rights campaign is that some species (notably great apes) are championed as candidates for rights thanks to their performance of what is conceived to be human-like characteristics—“a humanist framework that treats animals as liberal subjects” [96] (pp. 3–4). As legal scholar Maneesha Deckha explains, “The problematic outcome of focusing on those animals that humans recognize as similar to themselves is that all the others continue to be legally invisible and their exploitation justified” [12] (p. 87). Even Anne Peters, who largely supports the claims for animal rights, delivers a focused critique on human-modeled personhood [83] (p. 439). The historical analysis offered above largely supports these critiques. From the earliest encounters of West Europeans with the organs of hunted great apes to American laboratory tests for self-recognition on isolated primates, the inquiry into relations between the species has been driven by an unrelenting search for sameness. Scientific inquiries initially sought to locate the boundary in brain anatomy, while the twentieth century saw the forging of various linguistic and behavioral analogies between human and nonhuman primates, each episode redefining where the line falls.

But the human side of that line was simultaneously being redesigned while the various facets of life gained and lost significance. The debates over brain anatomy were consequential precisely because the brain had become the privileged site of human identity for nineteenth-century Englishmen. Language and speech became new boundary markers when the human was implicitly redefined as the speaking animal. The mirror test for self-recognition, in turn, became a criterion of inclusion at a moment when individuality and autonomy were taken to define a right-holding member of society. Furthermore, even if one accepts Deckha’s claim that in Western liberal legal systems, “personhood as a category would still be premised on a sharp mind/body split that exalts a certain type of reasoned cognition” [12] (p. 89), it is still crucial to analytically historicize the “cognition” that is being attributed or denied, tested, and debated. The history of science shows us the elasticity of the terms that are employed to characterize the mental state of humans and other animals, such as cognition and self-awareness. Therefore, when some animal rights movements adopt these terms to advance certain legal claims, they are not necessarily doomed to repeat all the biases of older legal systems. The flexibility of these terms is already being proven, for example, by the inclusion of the utterly alien minds of invertebrates in the legal concept of sentience [97].

When Jane Goodall reported back to Louis Leakey in the 1960 on her findings about chimpanzees’ tool use, he famously replied, “now we must redefine tool, redefine man, or accept chimpanzees as human” [98]. What the history of the simian mind shows is that the concepts of mind, the human, and the nonhuman animal were never discrete but were instead shaped through simultaneous and entangled processes of experimental, theoretical, and legal reconfiguration.

## Data Availability

The original contributions presented in this study are included in the article. Further inquiries can be directed to the corresponding author.
